# Expression of Tumor Necrosis Factor Receptor 2 Characterizes TLR9-Driven Formation of Interleukin-10-Producing B Cells

**DOI:** 10.3389/fimmu.2017.01951

**Published:** 2018-01-19

**Authors:** Olga Ticha, Lukas Moos, Harald Wajant, Isabelle Bekeredjian-Ding

**Affiliations:** ^1^Division of Microbiology, Paul-Ehrlich-Institut, Langen, Germany; ^2^Division of Molecular Internal Medicine, Department of Internal Medicine II, University Hospital Wuerzburg, Wuerzburg, Germany

**Keywords:** human, B cells, interleukin-10, tumor necrosis factor receptor 2, TLR9, Breg

## Abstract

B cell-derived interleukin-10 (IL-10) production has been described as a hallmark for regulatory function in B lymphocytes. However, there is an ongoing debate on the origin of IL-10-secreting B cells and lack of specific surface markers has turned into an important obstacle for studying human B regulatory cells. In this study, we propose that tumor necrosis factor receptor 2 (TNFR2) expression can be used for enrichment of IL-10-secreting B cells. Our data confirm that IL-10 production can be induced by TLR9 stimulation with CpG ODN and that IL-10 secretion accompanies differentiation of peripheral blood B cells into plasma blasts. We further show that CpG ODN stimulation induces TNFR2 expression, which correlates with IL-10 secretion and terminal differentiation. Indeed, flow cytometric sorting of TNFR2^+^ B cells revealed that TNFR2^+^ and TNFR2^−^ fractions correspond to IL-10^+^ and IL-10^−^ fractions, respectively. Furthermore, CpG-induced TNFR2^+^ B cells were predominantly found in the IgM^+^ CD27^+^ B cell subset and spontaneously released immunoglobulin. Finally, our data corroborate the functional impact of TNFR2 by demonstrating that stimulation with a TNFR2 agonist significantly augments IL-10 and IL-6 production in B cells. Altogether, our data highlight a new role for TNFR2 in IL-10-secreting human B lymphocytes along with the potential to exploit this finding for sorting and isolation of this currently ill-defined B cell subset.

## Introduction

The first observation of regulatory function of B cells producing interleukin-10 (IL-10) was demonstrated in mice with experimental autoimmune encephalomyelitis in 1996 by Janeway and colleagues (1). In the recent years, different subpopulations of IL-10-secreting B cells have been described in the mouse and their regulatory capacity has been demonstrated in models of infection and autoimmune diseases (2–5). However, in the human, very little is known on the role of suppressive B cells and their cellular origin. It was previously shown that a distinct subpopulation of B lymphocytes producing anti-inflammatory cytokines such as IL-10 could be differentiated from peripheral blood B cells *via* TLR9 stimulation with CpG DNA (6, 7). Furthermore, IL-10-secreting B cells were described in different types of infection including polyclonal B cell expansion triggered by *Staphylococcus aureus* (8), HIV patients (9, 10), and murine schistosomiasis models (11, 12). Various studies also indicated their reduced representation in peripheral blood of patients with autoimmune diseases and immune deficiencies (13–15).

Earlier, it was proposed that calcium-dependent signaling and vitamin D metabolism enhance or even enable IL-10 production in human peripheral blood B cells (7, 16–18). These molecular mechanisms seem well compatible with the finding that IL-10 production characterizes activated B cells undergoing differentiation to plasma blasts (19, 20). Notably, this finding also confirms earlier studies demonstrating that autocrine production of IL-10 increases plasma blast formation and Ig production (19, 21–23).

While IL-10 is a hallmark cytokine for immune suppression tumor necrosis factor (TNF) is a pleiotropic cytokine, which exists in two biologically active forms: cell-bound as a type II transmembrane protein and in a soluble variant derived thereof by proteolytic processing. TNF is primarily viewed as a cytokine enhancing immune defense against invading pathogens and mediating inflammation. As a consequence, TNF expression is tightly regulated (24–26) and its secretion can be selectively blocked in the context of endotoxin tolerance, which was recently proposed to impair microbial recognition and progression of periodontitis (27). Excessive and deregulated expression of TNF not only plays a crucial role in various autoimmune diseases including rheumatoid arthritis and Crohn’s disease but is also efficiently targeted in the clinic with various TNF-neutralizing drugs.

Tumor necrosis factor elicits its activities by stimulating two structurally related types of receptors, TNF receptor 1 (TNFR1) and tumor necrosis factor receptor 2 (TNFR2). TNFR1 (CD120a) is constitutively expressed on nearly all nucleated cell types, while expression of TNFR2 (CD120b) is limited to a subset of cell types of different origin including certain T lymphocyte subsets, thymocytes, cells of the myeloid lineage, specific neuronal subpopulations, endothelial cells, cardiac myocytes, and human mesenchymal stem cells (25, 28). TNFR1 is efficiently activated by both the soluble and the membrane-bound form of TNF, while TNFR2—despite high-affinity binding of soluble TNF—is only efficiently activated by membrane-bound TNF (29, 30).

The two TNF receptors play different roles in the context of an immune response and TNFR2 might contribute to later stages of the immune response and resolve inflammation rather than potentiating it. Indeed, signaling *via* TNFR2 has mainly been associated with proliferation, cytokine production, cell survival, differentiation, tissue repair, and angiogenesis, while TNFR1 contains an intracellular death domain that mediates strong activation of the highly proinflammatory classical NFκB pathway but also caspase activation and cell death (31–34). TNFR2 upregulation occurs under inflammatory conditions and could, thus, serve as a negative feedback mechanism to reduce cellular damage and danger signals generated by TNFR1 signaling. Indeed, soluble TNFR2 can capture TNF and prevent engagement of the proinflammatory receptor TNFR1 (35). Moreover, TNFR2 is highly expressed on T regulatory cells (Treg) and promotes the expansion and suppressive activity of this suppressive cell type (36–38). Additionally, TNF derived from conventional T cells supports Treg function in autoimmune diabetes and graft-versus-host disease (39, 40). Notably, these effects were found to be dependent on TNFR2 expression on Treg (41). For oncologists, TNFR2 has become an attractive target for dual suppression of TNFR2^+^ tumor cells and tumor-infiltrating Tregs, thus facilitating anti-tumor T cell responses and killing of malignant cells (42, 43). In this context, therapeutic inhibition of TNFR2 bears further potential since TNFR2 was identified as a myeloid-derived suppressor cell-promoting factor (44–47). In sum, these findings prompted us to ask whether TNFR2 might exert a similar role in regulatory B cells.

Considering the fact that TNFR2 expression has repeatedly been linked to IL-10 production (48, 49), this seemed an attractive hypothesis. However, while data from mice demonstrated a role of TNFR2 in B cell activation (50), in human B cells only scarce information was available. The published data suggested redundant roles of TNFR2 and CD40 in B cell activation based on the common signaling pathway involving TRAF2 (51). Moreover, in patients, TNF-targeting therapies have been associated with increased development of autoantibodies and lupus-like syndromes (52–54). Albeit these clinical observations are not well understood, they indicate that TNF possesses a so far not acknowledged regulatory role in B cell differentiation. Since expression patterns and function of TNFR2 in B cells remain largely unexplored, we opted to investigate a possible association of TNFR2 with development and function of IL-10-secreting B cells. Here, we present original data showing that TNFR2 expression in B cells is stimulated *via* TLR9 and coincides with IL-10 release and terminal B cell differentiation.

## Materials and Methods

### Cells

Peripheral blood mononuclear cells (PBMC) were isolated from buffy coats from healthy donors obtained from German Red Cross South institute for transfusion medicine and immune hematology (Frankfurt am Main, Germany). The use was approved by the ethics committee from the medical faculty of the University of Frankfurt, Germany (Approval #154/15). PBMC were isolated by Pancoll gradient centrifugation (PAN-Biotech, Aidenbach, Germany) followed by B cell positive selection with anti-CD19 microbeads (Miltenyi Biotech, Bergisch-Gladbach, Germany) according to the manufacturer’s protocol. For plasmablast enrichment, CD138 MicroBeads (Miltenyi Biotech) were applied. Purity was controlled by flow cytometry and was ≥97%. Isolated cells were cultivated in RPMI 1640 (Gibco, Life Science, Darmstadt, Germany) supplemented with 10% FCS (Sigma-Aldrich Chemie GmbH, Munich, Germany), 1% penicillin/streptomycin, 1% l–glutamine, and 1% HEPES buffer (all from Biochrom, Berlin, Germany). Cells were seeded at a concentration 10^6^ cells/ml (if not stated differently) and cultivated in 96-well plates (Greiner CELLSTAR^®^ round bottom 96-well plates; Greiner Bio-One, Kremsmünster, Austria). All cells were cultivated in a 5% CO_2_ incubator at 37°C. For stimulation, 1 µM full-length PTO modified CpG 2006 (5′-tcgtcgttttgtcgttttgtcgtt-3′) purchased from Eurofins MWG Biotech (Munich, Germany) was applied. TNC-scTNF(143N/145R), a highly active nonameric human TNF mutant with specificity for TNFR2 as described elsewhere (30). It was used at a concentration of 100 ng/ml. All experiments were performed in technical duplicates.

### Cell Sorting

B cell subpopulations were sorted on a FACSAria™ Fusion (BD Biosciences, Heidelberg, Germany) using the version 8.0.1 of the BD FACS Diva software. Purity of sorted subpopulations was confirmed by remeasuring of samples. Sorted cells were washed, counted, and checked for viability using trypan blue (Applichem Panreac, Darmstadt, Germany). TNFR2-positive and -negative B cells were sorted from total B cells stimulated for 2 days by 1 µM CpG ODN 2006, if not stated otherwise.

### Flow Cytometry

Phenotypic analysis of human B cell subsets was performed with the following antibodies: anti-CD19-PE-Cy7 (Beckman Coulter, Marseille, France), anti-CD27-BV421 (BD Biosciences, Heidelberg, Germany), anti-IgM-PerCP/Cy5.5 (BioLegend, CA, USA), anti-IgM-BV605 (BioLegend), anti-CD38-PE (BD Biosciences), anti-TNFR1(CD120a)-FITC (Miltenyi Biotech), anti-TNFR2(CD120b)-APC (R&D Systems, Inc., Minneapolis, MN, USA), and murine IgG_2A_-APC (R&D Systems, Inc.) as isotype control where indicated. Cells were incubated in the dark for 30 min at 4°C in PBS with 0.5% FCS. Samples were acquired using a FACS LSRII SORP (BD Biosciences, Heidelberg, Germany), and cytometry data (LMD files) were analyzed with Kaluza software (Beckman Coulter). The aqua fluorescent reactive dye (LIVE/DEAD Fixable dead Cell stain Kit, Invitrogen, CA, USA) was used for definition of live and dead cells.

For staining of IL-10-producing B cells we used the IL-10 Secretion Assay (Miltenyi Biotech, Bergisch-Gladbach, Germany). B cells were stimulated for 40 h in culture medium with 1 µM CpG ODN 2006. Staining with anti-IL-10 was performed according to the protocol provided by the manufacturer with a prolonged incubation of cells labeled with IL-10 catch reagent (6 h) in presence of 0.25 µM CpG for restimulation. Cells were subsequently stained for expression of other surface markers before measurement on a flow cytometer.

### ELISA

Supernatants were collected from cells at the indicated time points. IL-10 and IL-6 were quantified by ELISA (human IL-10 and IL-6 ELISA OptEIA Sets, BD Bioscience, Heidelberg, Germany). Human immunoglobulins were quantified using Human IgG/IgM/IgA ELISA Quantitation Sets (all from Bethyl Laboratories, TX, USA).

### ELISpot and FluoroSpot Assays

For ELISPOT assays, experiments were performed in 96-well MultiScreen HTS IP plates (0.45 µm, clear, Merck Chemicals GmbH, Darmstadt, Germany), coated with capture antibody (monoclonal antibody to human IgG MT91/145; MabTech, Stockholm, Sweden) in DPBS overnight at 4°C. On the following day, the ELISpot plate was washed three times with PBS and blocked for 2 h at room temperature with culture medium before cells were seeded. After 20 h, incubation cells were discarded and plates washed with PBS. Biotinylated anti-human IgG detection antibody (MT78/145; MabTech) was added in PBS with 10% FCS and plates incubated for 2 h at room temperature. After washing in PBS with 0.05% Tween20 (Sigma-Aldrich Chemie GmbH, Munich, Germany), alkaline phosphatase (AP)-conjugated Streptavidin (BD Biosciences) was added 1:1,000 in PBS with 10% FCS followed by incubation for 1 h. Development of the plate was performed with the AP conjugate substrate kit (Bio-Rad Laboratories GmbH, München, Germany) and the reaction was stopped with water and the plate dried overnight.

Human IL-10 FluoroSpot^BASIC^ (550) (MabTech) was used for enumeration of IL-10-secreting B cells. The plate was coated with capture antibody in PBS overnight at 4°C, washed three times, and blocked for 2 h before seeding of cells with culture medium supplemented with 10% FCS. After 4 days of cultivation, cells were removed, the plate was washed five times with PBS, and the development of the assay was performed according to the manufacturer’s protocol. Spots from enzymatic and fluorescence assays were quantified with an iSpot FluoroSpot Reader System (AID, Strassberg, Germany).

### Proliferation Assay

Proliferation was assessed using CFSE staining. CD19^+^ isolated B cells were stained in 1 µM solution of CFSE (eBioscience, San Diego, CA, USA) for 10 min at room temperature, staining was stopped with FCS, and cells were washed three times with cold RPMI containing 10% FCS in a pre-cooled centrifuge to remove unbound CFSE. Stained cells were seeded 1 × 10^6^/ml and stimulated for 4 days before quantification of CFSE dilution by flow cytometry.

### Statistical Analysis

Statistical analysis of results was carried out using GraphPad Prism 7.01 (Graphpad Software Inc., San Diego, CA, USA). Data were analyzed using paired two-tailed Student’s *t*-test. A standard level of statistical significance α = 0.05 was used. Symbols representing *p* values are used as follows: *p < 0.05, **p < 0.01.

## Results

### Stimulation with CpG ODN Induces Expression of TNFR2 on Human Peripheral Blood B Cells

**Figure 1:**
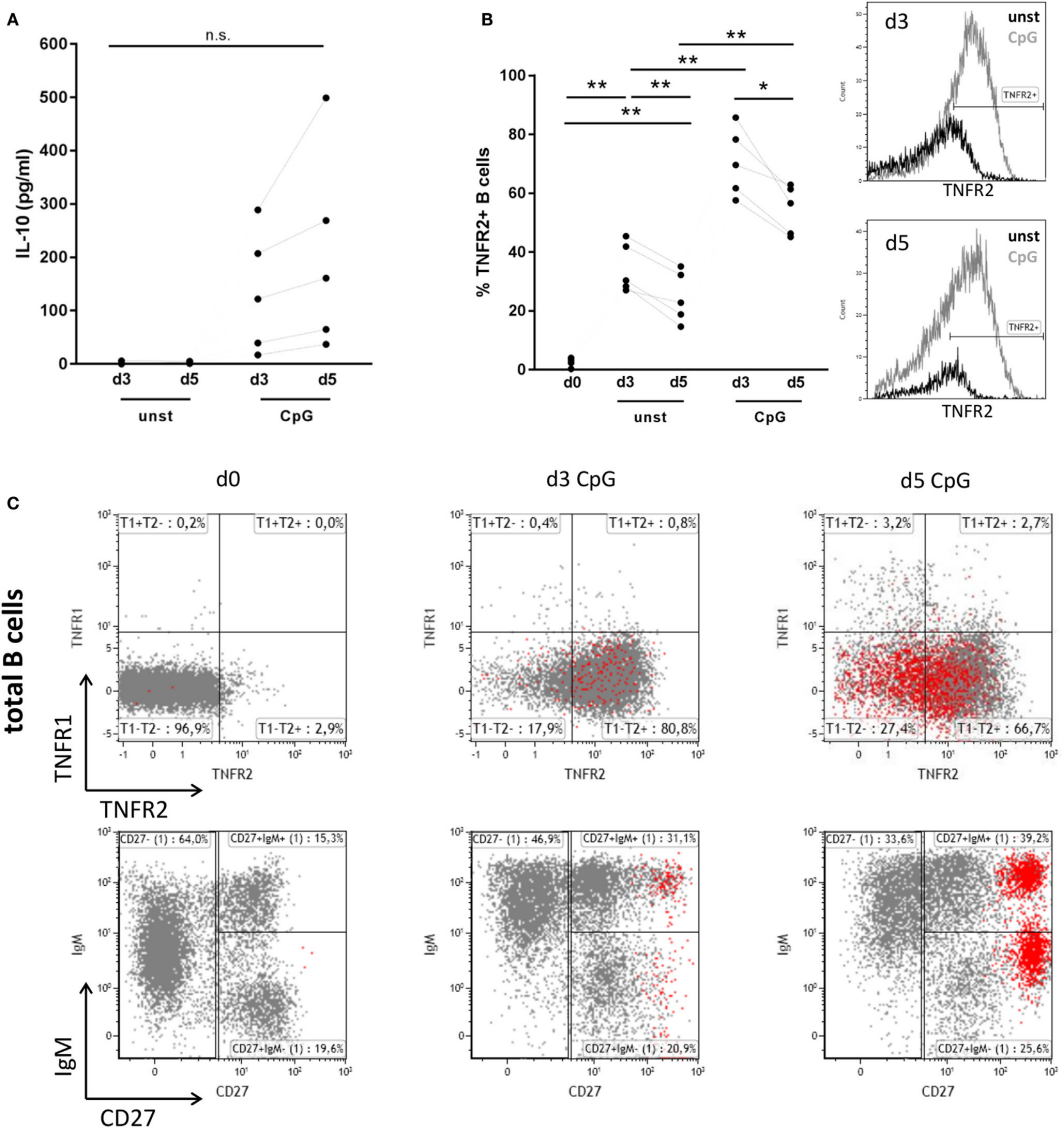
Interleukin-10 (IL-10) production, tumor necrosis factor receptor 2 (TNFR2), and tumor necrosis factor receptor 1 (TNFR1) expression after CpG ODN stimulation of B cells. Freshly isolated B cells (d0) and on days 3 and 5 after stimulation with CpG ODN were studied for **(A)** IL-10 production in supernatants (*n* = 5 independent donors) and **(B)** TNFR2 expression on unstimulated and stimulated B cells (*n* = 5 independent donors). The histograms for TNFR2 expression are shown as an overlay on days 3 and 5 with marker placed based on level of isotype control fluorescence. The graph summarizes the results from *n* = 5 independent donors (*p < 0.05, **p < 0.01, n.s., not significant). **(C)** TNFR1 and TNFR2 expression on total CD19^+^ B cells was analyzed by flow cytometry in freshly isolated B cells (d0) and on days 3 and 5 after stimulation with CpG ODN. CD38^high^ CD27^high^ plasma blasts are highlighted in red. The lower panel shows the phenotype profile of CpG-stimulated B cells based on IgM and CD27 markers. Results from one representative of four independent donors are depicted.

As previously described IL-10 release from human peripheral blood B cells can be elicited by stimulation of TLR9 with CpG ODN. When compared to release of IL-6, secretion of IL-10 was previously found to be delayed, indicating that B cell differentiation might represent a prerequisite for IL-10 synthesis (6, 7). Here, we confirm that stimulation of human B cells with CpG ODN enables high release of IL-10 when compared to unstimulated B cells where IL-10 levels lay below the detection threshold of the ELISA (Figure 1A). Concomitant analysis of B cell expression of TNFR2 revealed that—similarly to IL-10—TNFR2 surface expression gradually increased after stimulation (Figure 1B). Albeit TNFR2 was detectable on surviving unstimulated cells expression levels were significantly lower (Figure 1B). These observations prompted us to ask whether TNFR2 expression is associated with cell survival and IL-10 secretion and could, possibly, serve as a marker characterizing IL-10-producing B cells. To this end, we used TLR9 ligand CpG ODN 2006 as a tool to study proliferation and the differentiation process into plasmablasts.

Analysis of expression of TNFR1 and TNFR2 in freshly isolated human B cells revealed that surface expression of both receptors is nearly absent or low, respectively (Figure 1C). However, stimulation of B cells with TLR9 ligand CpG ODN induced the expression of TNFR2, while expression of TNFR1 remained low and contained to a small and circumscribed B cell population (Figure 1C). Of note, only few B cells highly positive for both TNF receptors were detected. Interestingly TNFR2 expression on B cell surface was reduced on arising CD38^high^ CD27^high^ B cells corresponding to plasma blasts on day 5 (Figure 1C).

**Figure 2:**
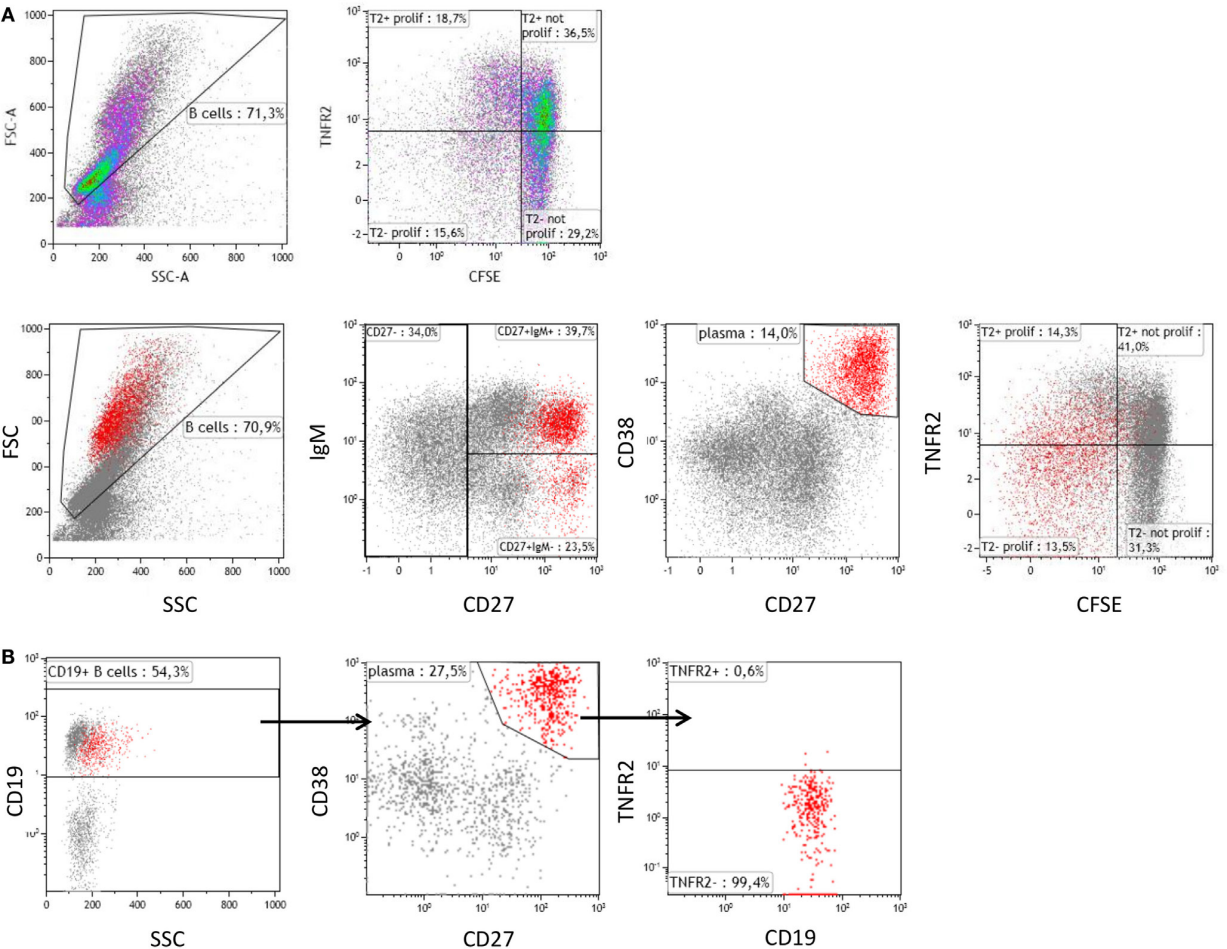
Tumor necrosis factor receptor 2 (TNFR2) expression on proliferating B cells and plasma cells. **(A)** On day 4 after stimulation with CpG ODN, TNFR2 expression was analyzed on proliferating B cells and plasma blasts (CD38^high^ CD27^high^ highlighted in red). B cell proliferation was visualized by CFSE dilution. Data from one representative donor of four are shown. FSC: Forward scatter; SSC: Side scatter. **(B)** Plasma cells were enriched by isolation of CD138^+^ cells from peripheral blood and stained for TNFR2 expression. The results obtained from one representative of four independent donors are provided.

Further analysis revealed that CpG-induced TNFR2 expression was high in proliferating B cells stained by CFSE (Figure 2A). Again, loss of TNFR2 expression was observed in developing plasma blasts (CD27^high^ CD38^high^) (Figure 2A). Notably, TNFR2 expression was absent on CD138^+^ plasma cells freshly isolated from PBMC (Figure 2B).

**Figure 3:**
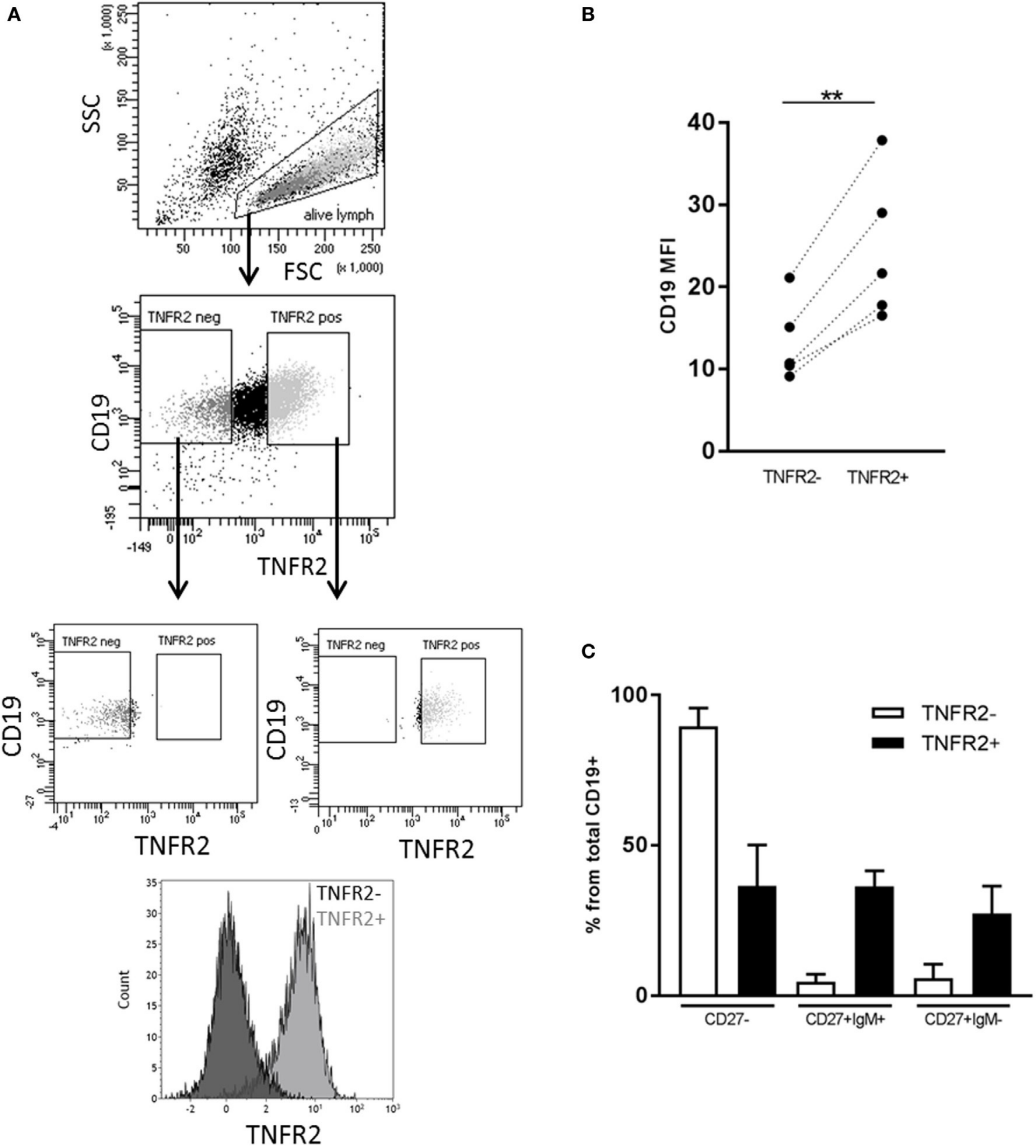
Representation of B cell subsets in sorted TNFR2^+^ and TNFR2^−^ B cell fractions. **(A)** Gating and sorting strategy for obtaining TNFR2^+^ and TNFR2^−^ B cells. FSC: Forward scatter; SSC: side scatter. **(B)** Left: mean fluorescence intensity (MFI) for CD19 expression is compared on TNFR2^−^ and TNFR2^+^ B cells. Results from *n* = 5 donors are shown (**p < 0.01). **(C)** Distribution of B cell subpopulations in TNFR2^−^ and TNFR2^+^ sorted B cell fractions. The percentage of cells in CD27^−^, CD27^+^ IgM^+^, and CD27^+^ IgM^−^ subpopulations from total B cells is shown as mean values ± SD of *n* = 4 donors. B cells subpopulation’s distribution in TNFR2^+^ and TNFR2^−^ sorted population is statistically different (two-way ANOVA *p* < 0.0001).

Next, we sought to identify the B cell subsets expressing TNFR2. To this end, we sorted TNFR2^+^ and TNFR2^−^ B cells on day 2 after stimulation with CpG ODN and analyzed the composition of B cell subpopulations in the CpG ODN-responsive fractions (see Figure 3A for sorting scheme). Notably, expression of CD19 was higher in the TNFR2^+^ subpopulation, most likely reflecting a higher activation status of these cells (Figure 3B). Furthermore, IgM^+^ and class-switched memory B cells were predominantly found in the TNFR2^+^ fraction while CD27^−^ B cells (naïve and transitional B cells) were detectable in both fractions, the TNFR2^−^ fraction consisting of >80% CD27^−^ B cells (Figure 3C).

### TNFR2-Expressing B Cells Develop into Antibody-Secreting Cells

**Figure 4:**
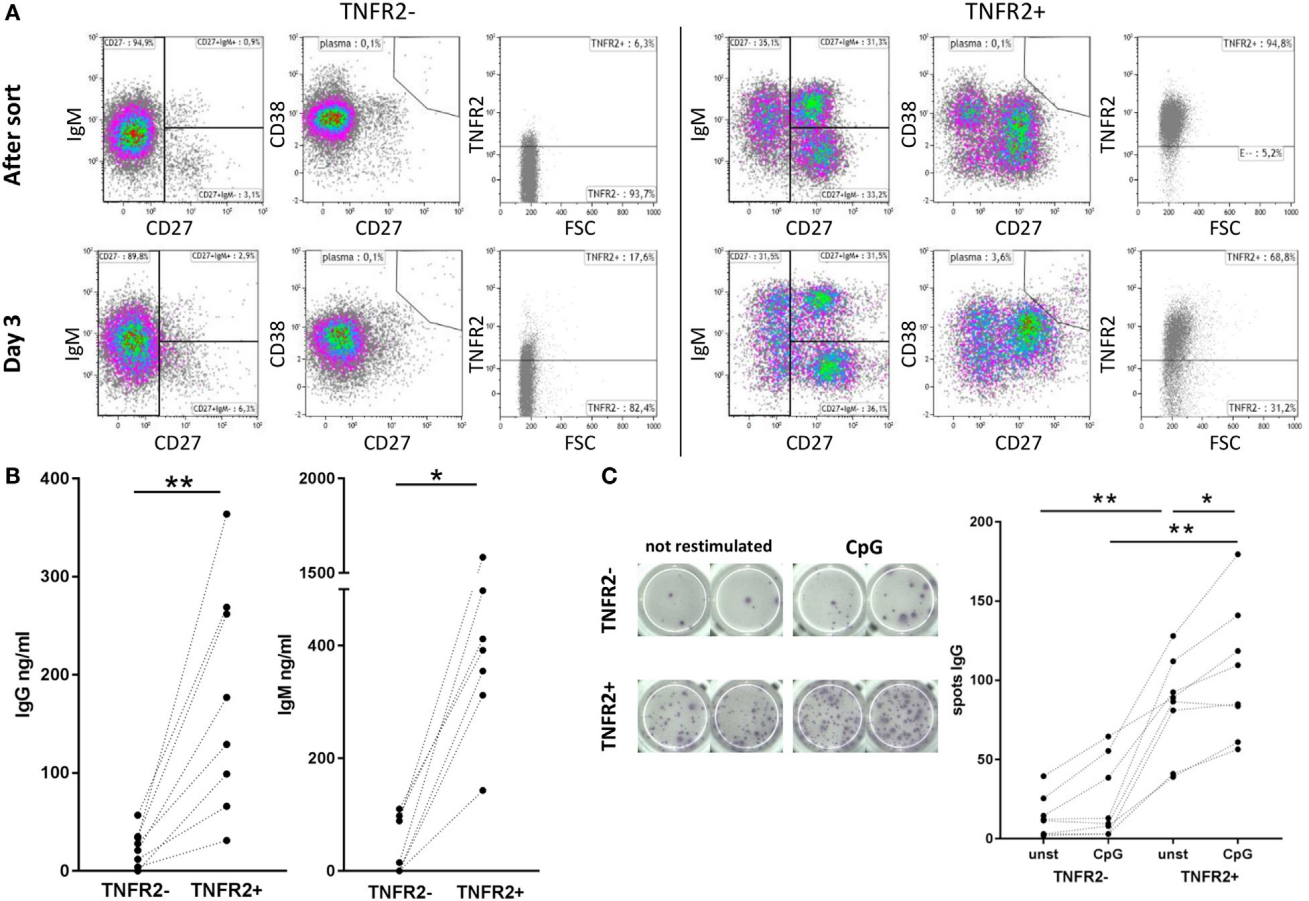
Differentiation and immunoglobulin production of CpG ODN-induced TNFR2^+^ B cells. **(A)** Flow cytometric analysis of B cell phenotypes in TNFR2^−^ and TNFR2^+^ B cells was performed immediately after sort and after three additional days in culture without restimulation. Cells were stained for TNFR2, IgM, CD27, and CD38. Results from one representative donor are shown. FSC: forward scatter. **(B)** IgG and IgM production was quantified in supernatants from TNFR2^−^ and TNFR2^+^ B cells on day 4 after sort without restimulation. The graphs depict results from *n* = 8 donors and *n* = 7 donors, respectively (*p < 0.05, **p < 0.01). **(C)** For visualization of IgG-secreting cells by ELISPOT sorted TNFR2^−^ and TNFR2^+^ B cell fractions were cultured for 4 days with and without restimulation with CpG ODN. On day 4, B cells were washed, seeded at 15 × 10^3^ B cells/well on the ELISPOT membrane, and incubated overnight. One representative experiment is shown in the left panel; the results obtained from *n* = 8 donors are shown in the graph on the right (*p < 0.05, **p < 0.01).

Phenotypical analysis of day 2-sorted TNFR2^+^ and TNFR2^−^ B cells was repeated after 3 days of cell culture in the absence of restimulation. The results showed that TNFR2^−^ B cells mainly consisted of IgM^+^ CD38^+^ CD27^−^ naïve B cells (Figure 4A, left panel). This population remained unchanged over the culture period. On the contrary, the TNFR2^+^ fraction contained class-switched and IgM^+^ memory B cells next to naïve (IgM^+^ CD27^−^ CD38^+/−^) B cells (Figure 4A, right panel). The proportional representation was unaltered after the 3-day culture. However, when supernatants of sorted TNFR2^+^ and TNFR2^−^ B cells were probed for Ig secretion, we found that TNFR2^+^ B cells secrete large quantities of IgM (143–1584 ng/ml) and IgG (31–364 ng/ml) (Figure 4B). These findings supported the concept that TNFR2^+^ B cells contain developing plasmablasts. Confirming this, Figure 4C visualizes the formation of antibody-secreting cells *via* IgG ELISPOT. Notably, TNFR2^+^ B cells released IgG without restimulation but restimulation with CpG ODN further increased the number of IgG-secreting cells (Figure 4C).

### TNFR2 Expression Correlates with IL-10 Production

**Figure 5:**
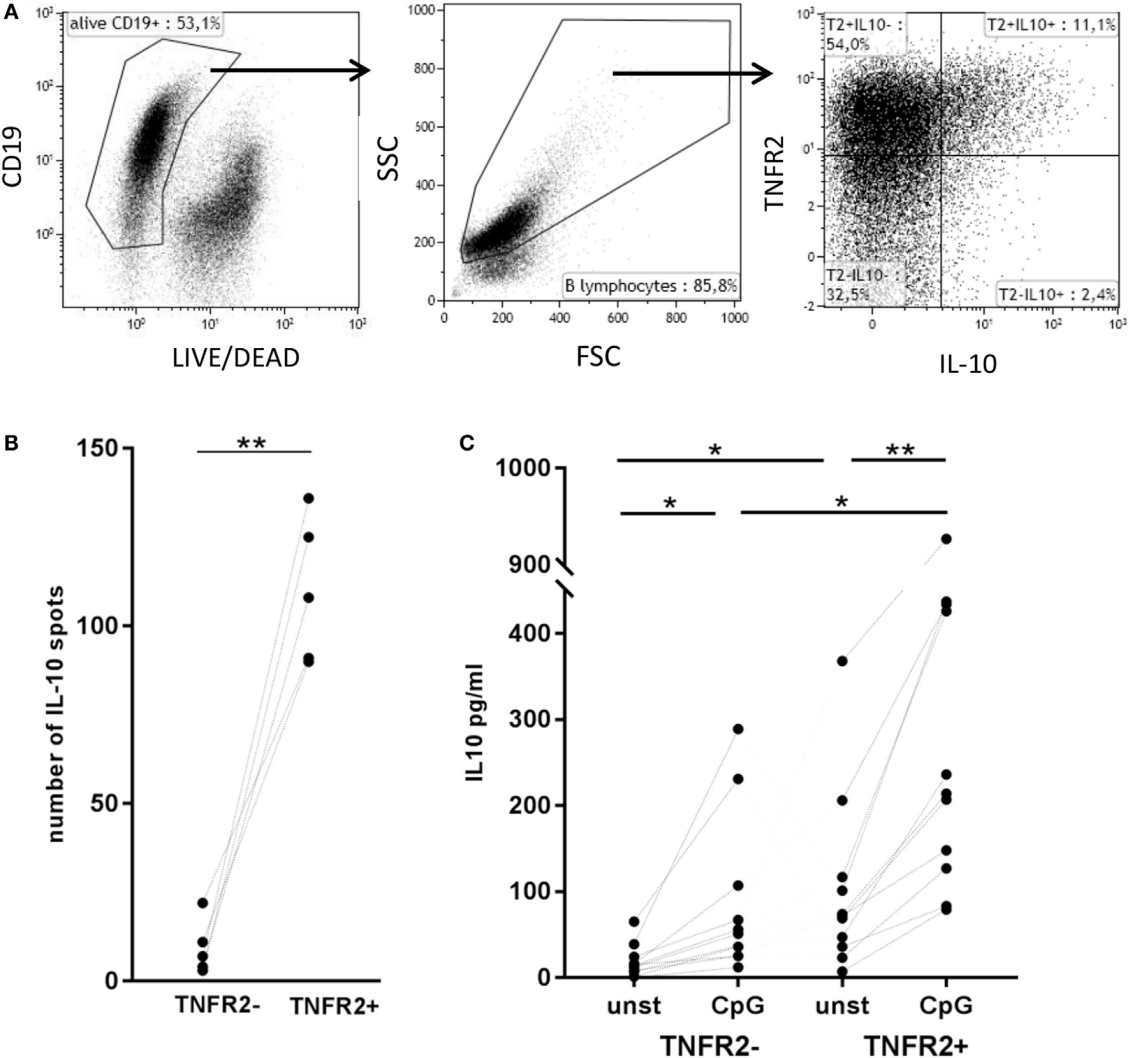
Correlation of tumor necrosis factor receptor 2 (TNFR2) expression with interleukin-10 (IL-10) secretion. **(A)** Flow cytometric detection of TNFR2 on the surface of IL-10-secreting cells on day 2 after CpG ODN stimulation. One representative experiment of *n* = 3 independent experiments is shown. FSC: forward scatter; SSC: side scatter. **(B)** Quantification of IL-10-producing B cells in TNFR2^−^ and TNFR2^+^ B cell fractions (30 × 10^3^ cells/well) was achieved by FluoroSpot analysis after 4 days of culture in the absence of restimulation. The results of *n* = 5 independent donors are shown (**p < 0.01). **(C)** IL-10 production was measured in 4 day supernatants of sorted TNFR2^−^ and TNFR2^+^ B cells. Sorted B cells were either left unstimulated or restimulated with CpG ODN. The graph depicts the results obtained in *n* = 10 independent donors (*p < 0.05, **p < 0.01).

Next, we asked whether TNFR2 expression correlates with IL-10 secretion. Flow cytometric analysis showed that B cells secreting IL-10 are, indeed, TNFR2 positive (Figure 5A). Using this type of analysis they form a subpopulation of the TNFR2^+^ B cells. Sorting of TNFR2^+^ and TNFR2^−^ fractions further showed that IL-10 secreting cells are located in the TNFR2^+^ fraction and nearly absent in the TNFR2^−^ fraction (Figure 5B). Upon restimulation of both fractions with CpG ODN, IL-10 secretion became detectable in the supernatants of TNFR2^−^ B cells but the increase was significantly higher in TNFR2^+^ B cells (Figure 5C). Altogether, these data indicate that TNFR2-negative B cells are less prone to secrete IL-10 on a per cell basis.

### IgM Memory B Cells Represent the Major Source of IL-10

**Figure 6:**
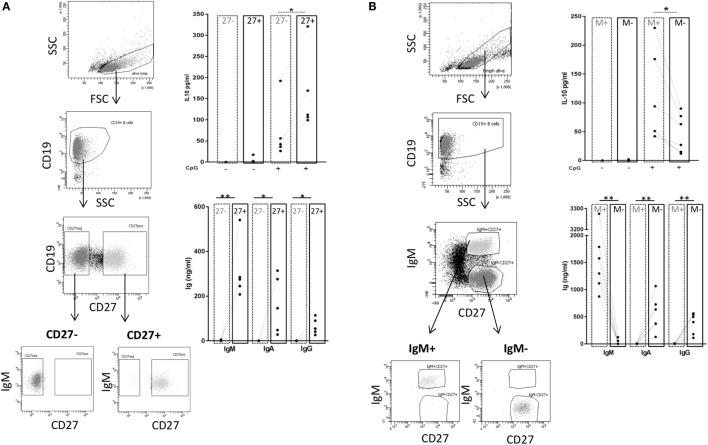
TLR9-dependent interleukin-10 (IL-10) and Ig release in sorted B cell subpopulations. Gating and sorting strategies used for obtaining CD27^−^ and CD27^+^ **(A)** or IgM^+^ memory versus class-switched memory B cells **(B)** are shown in the left panels. IL-10 and IgM, IgA, and IgG production were quantified in B cell supernatants on day 3 from **(A)** 2 × 10^5^ cells/well CD27^−^ (dotted frames) and CD27^+^ B cells (full frames) from *n* = 5 donors; and from **(B)** 1 × 10^5^ cells/well IgM memory B cells (M^+^ = IgM^+^ CD27^+^; dotted frames) and switched memory B cells (M^−^ = IgM^−^ CD27^+^; full frames) isolated from *n* = 6 (IL-10) and *n* = 5 donors (Ig).

**Figure 7:**
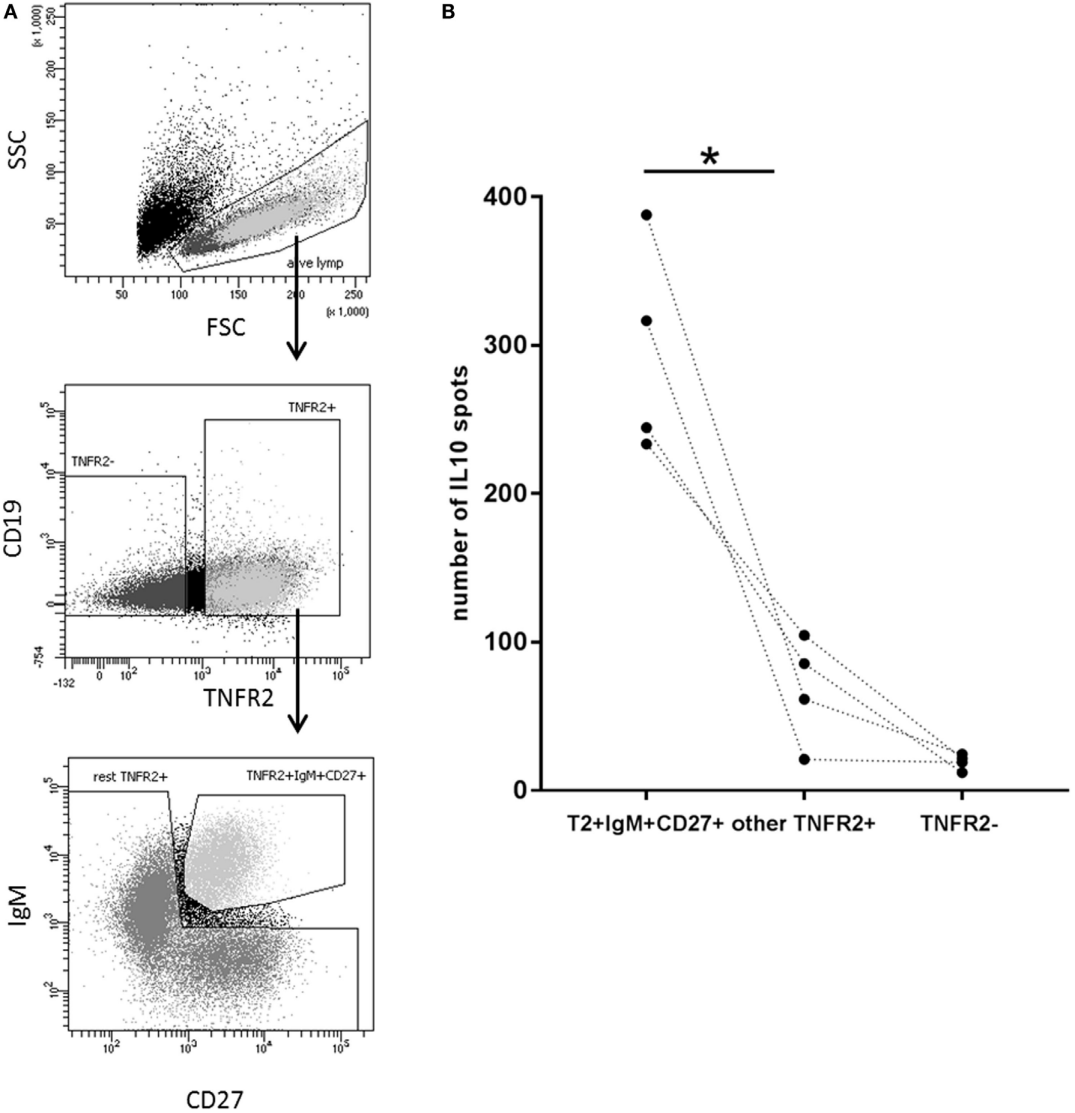
Identification of interleukin-10 (IL-10)-producing B cell subsets in the TNFR2^+^ B cell fraction. **(A)** Gating and sorting strategies used after 1-day stimulation of total B cells with CpG ODN for obtaining TNFR2^+^ IgM^+^ CD27^+^, residual TNFR2^+^, and TNFR2^−^ B cells subpopulations. FSC: forward scatter; SSC: side scatter. **(B)** After four additional days at 25 × 10^3^ cells/well, IL-10-producing cells were enumerated using FluoroSpot. The graph summarizes the results from *n* = 4 independent donors (*p < 0.05).

Next, we wanted to investigate whether there is a B cell subset specifically characterized by IL-10 production. To this end, we sorted CD27^+^ and CD27^−^ B cell fractions (see Figure 6A, left panel for sorting scheme). As previously described, CpG ODN stimulation triggered IgM, IgG, and IgA secretion in CD27^+^ B cells but not in CD27^−^ B cells (Figure 6A, right panel) (55, 56). IL-10 was induced in both fractions but levels were significantly higher in the CD27^+^ fraction (Figure 6A, right panel). Further sorting of the memory B cell population, e.g., IgM^+^ versus class-switched (IgM^−^) memory B cells (see Figure 6B, left panel for sorting scheme), revealed that albeit CpG ODN-induced IL-10 was detectable in both fractions significantly higher levels were obtained in the IgM^+^ CD27^+^ B cell fraction (Figure 6B, right panel). Finally, we sorted three more CpG ODN-responsive fractions: (1) TNFR2^+^ IgM^+^ CD27^+^, (2) residual TNFR2^+^, and (3) TNFR2^−^ B cells. The results confirmed that TNFR2^+^ IgM^+^ memory B cells represent the major source of IL-10 (Figure 7).

### TNFR2 Is Functionally Active on B Cells

**Figure 8:**
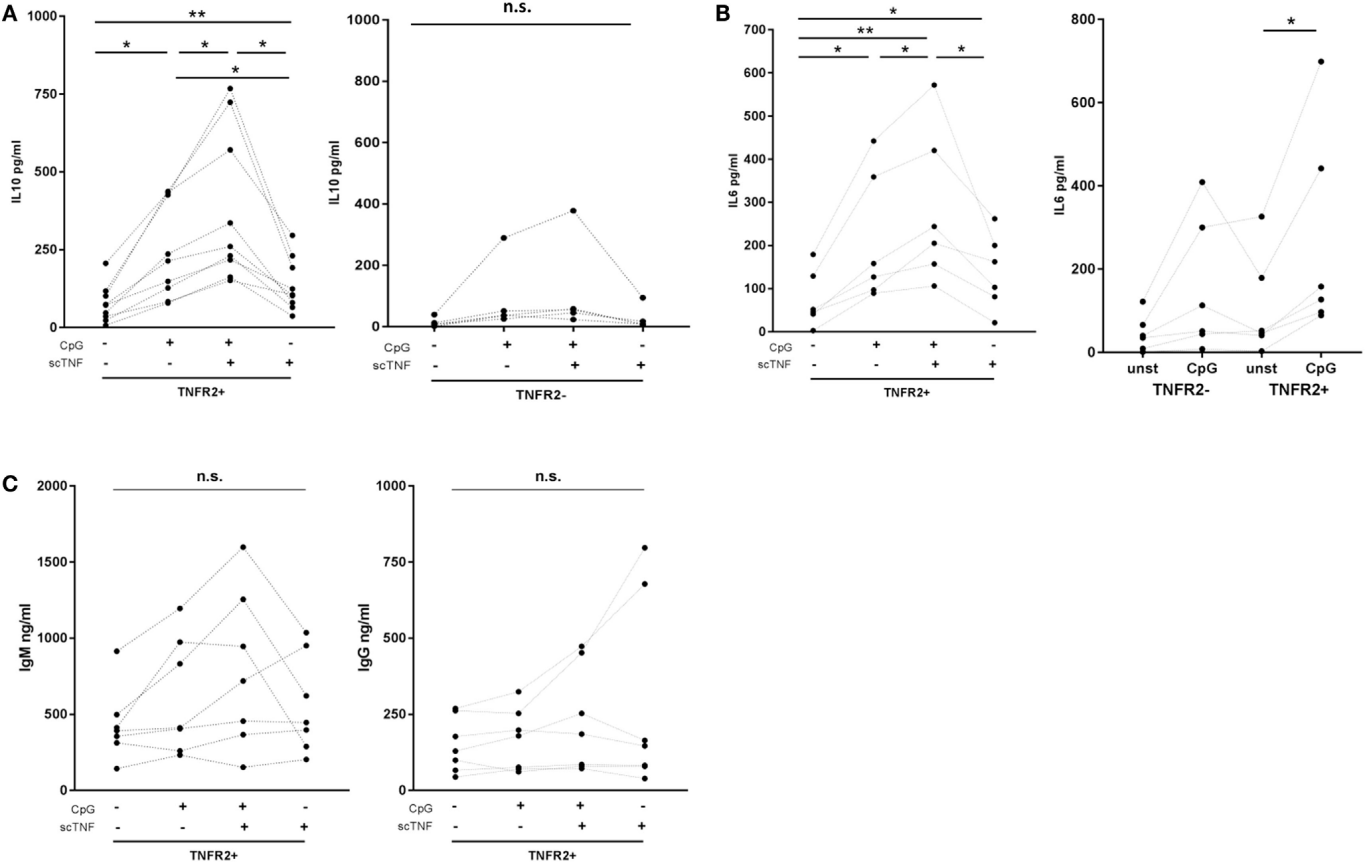
Functional impact of TNFR2. Sorted TNFR2^−^ and TNFR2^+^ B cell fraction were either left unstimulated or restimulated with CpG ODN for 4 days. For stimulation of TNFR2, experiments were carried out in the presence or absence of agonist TNC-scTNF(143N/145R) (scTNF). Cytokine [interleukin-10 **(A)**, IL-6 **(B)**] and Ig [**(C)**; IgM left panel, IgG right panel] secretion were measured in the supernatants with ELISA. Results from individual donors are depicted by the lines connecting the single values obtained (*p < 0.05, **p < 0.01, n.s., not significant).

Finally, we asked whether TNFR2 expressed on B cells is functional. To selectively stimulate TNFR2, we used TNC-scTNF(143N/145R), a nonameric variant of the TNFR2-specific TNF mutant TNF(143N/145R) (57), which mimics the membrane-bound trimeric form of TNF (30, 58). TNC-scTNF(143N/145R) was added to the TNFR2^+^ B cells stimulated with CpG ODN for 2 days. B cells were kept in culture for additional 4 days and supernatants collected and analyzed for cytokine and Ig production. The results obtained showed that IL-10 production is significantly increased in the presence of TNC-scTNF(143N/145R), which was even more accentuated if B cells were restimulated with CpG ODN (Figure 8A). No relevant effect was observed when the TNFR2 agonist was added to TNFR2^−^ B cell cultures, thus confirming its specificity. Similarly, in TNFR2^+^ B cells, IL-6 secretion was increased by TNC-scTNF(143N/145R), independent of the restimulation with CpG ODN (Figure 8B). Analysis of IgM and IgG secretion in the presence and absence of TNC-scTNF(143N/145R) revealed high donor variability; no statistically significant alteration could be attributed to TNFR2 stimulation (Figure 8C).

## Discussion

In this study, we investigated whether expression of TNFR2 could be used to characterize and purify human IL-10-secreting B cells. Our results show that in human B lymphocytes, upregulation of TNFR2 expression coincides with IL-10 production. Reasoning that sorting of IL-10-positive B cells cannot be based on IL-10 secretion and that stimulation of the BCR with anti-IgM should be avoided TNFR2 might represent an option for identification and sorting of IgM^+^ CD27^+^ IL-10^+^ B cells, thus avoiding unnecessary manipulation the BCR.

A previous study reported that expression of TNFR1 and TNFR2 is limited to 3 or 10%, respectively, of peripheral CD19^+^ human B cells (59). In this study, we confirmed that TNF receptor expression is nearly absent in unstimulated B cells (Figure 1C). However, we found that in analogy to other cell types, TNFR2 expression is inducible while TNFR1 expression is constitutive but weak and only altered on a small B cell subset upon TLR9 stimulation (Figure 1C). Notably, the presence of both receptors on stimulated human B cells was described in an early study using anti-μ or *S. aureus* Cowan strain I for B cell activation (60).

In this study, we investigated whether expression of TNFR2 would be confined to defined B cell subpopulations. Previous studies demonstrated that CpG ODN induce vigorous proliferation of IgM^+^ B cells that were found to belong to the memory (CD27^+^) B cell fraction (56, 61–63). In our experiments, the B cells remaining negative for TNFR2 expression upon CpG ODN stimulation belonged to the naïve B cell fraction (Figure 3C), while induction of TNFR2 expression was observed on both naïve and memory B cell subsets (Figure 3C). Nevertheless, we found that TNFR2 expression correlates with B cell proliferation and initial differentiation (Figure 2). It is, however, reduced and finally lost upon terminal differentiation into plasma blasts (Figures 1C and 2A). In accordance, TNFR2 is not expressed on plasma cells isolated from human peripheral blood (Figure 2B).

The expression kinetic of TNFR2 coincided with CpG ODN-induced IL-10 production, which reaches a maximum after approximately 48 h (7). This observation is well in line with the concept that IL-10-secreting B cells arise as an intermediate differentiation stage during terminal B cell differentiation (19, 20) and it was further supported by the finding that the amount of IgM and IgG measured in the supernatants of TNFR2^+^ B cells was significantly higher than that detected in TNFR2^−^ B cells (Figure 4B). Similarly, Ig-secreting cells were predominantly detected in the TNFR2^+^ B cell fraction (Figure 4C).

In macrophages, inducibility of TNFR2 expression by TLR9 stimulation was suggested to be mediated by PKB/Akt signaling (64). Previous work from our group and others highlighted the central role of the PKB/Akt pathway in both induction of TLR-dependent IL-10 release (27, 49) and in mediating TLR9-induced B cell effector function including survival, proliferation, cytokine secretion, and differentiation (7, 56). Due to its key role in CpG ODN-triggered B cell proliferation and differentiation, it is, thus, very likely that PKB/Akt signaling also represents a prerequisite for induction of TNFR2 expression in human B cells.

Not surprisingly, origin and development of B regulatory cells are subjects of intense debate. According to the literature, IL-10 is produced in a wide range of B cell subpopulations defined by numerous markers but, to date, no common surface marker has been discovered (65). In this study, we demonstrated that TNFR2 can be used as a cell surface marker for sorting of human IL-10-secreting B cells (Figure 5B). Interestingly, TNFR2^−^ B cells remain responsive to CpG ODN stimulation and secrete IL-10 in response to restimulation of TLR9 (Figure 5C). However, IL-10 secretion levels are markedly higher in unstimulated TNFR2^+^ B cells and increase further upon restimulation (Figure 5C). Altogether, these data reveal that the IL-10 response is characterized by a certain degree of plasticity, a finding well compatible with an intermediate stage in a process of cellular differentiation.

To date, it is well accepted that B cells producing IL-10 are involved in the regulation and termination of immune responses and that the suppressive effect of IL-10 inhibits antigen presentation and cytokine production by myeloid cells as well as Th1 and Th2 polarization (66–68). It was, therefore, important to prove that TNFR2 is functional on human B cells. Here, we used the TNFR2 agonist TNC-scTNF(143N/145R) (30, 58) to assess the functional impact of TNFR2 on TLR9-mediated B cell activation. Our data revealed that stimulation of TNFR2 augmented IL-10 and IL-6 secretion (Figure 8), two cytokines vital for plasma cell differentiation (19, 21, 69, 70). This effect was accentuated after restimulation of TNFR2^+^ B cells with CpG ODN (Figure 8). These findings also corroborated the observations by Hostager et al. who described a role of TNFR2 in human B cells stimulated through CD40 (50). However, in contrast to this earlier report, we did not observe a relevant effect on Ig production (Figure 8). Nevertheless, the increased cytokine concentrations could enhance terminal differentiation *via* autocrine feedback.

In Tregs, TNFR2 was shown to enhance survival and proliferation (71–73). Additionally, signaling *via* TNFR2 induces an NFκB-dependent transcriptional program that promotes suppressive activity of Treg *via* enhancement of FoxP3 expression (37, 74, 75). However, the only additional information published on B cells is that interaction of human B cells with membrane-bound TNF presented on activated CD4^+^ T cells serves as a costimulatory signal for B cell activation in IL-4-mediated IgG_4_ and IgE production (76). Thus, it can be only speculated that co-stimulation of TNFR2 by T cells or monocytes carrying membrane-bound TNF (26) could influence other B cell functions including suppressive capacity. Follow-up studies will need to explore the activated signaling pathways and the emerging functional properties.

It has further been described that TNFR2^+^ Tregs are elevated in the blood of asymptomatic malaria patients (77, 78). In this disease state, they might play a role in control of disease manifestation. Interestingly, polyclonal B cell activation accompanies many types of infections and results in the expansion of IL-10-releasing B cells (8). At present, this is thought to represent an immune evasion mechanism that prevents pathogen recognition by T- and B cells (79). However, similarly to Treg in malaria, the role of IL-10-secreting B cells in infection could also consist in limiting the inflammatory reaction to the infecting microbe. Future work will have to prove this hypothesis and confirm TNFR2 expression on pathogen-activated B cells.

Despite the limitations of the experimental system used, e.g., T cell-independent and antigen-independent stimulation of B cells with CpG ODN, the results of this study highlight the potential of TNFR2 to serve as a marker identifying human IL-10-secreting B cells in infection and autoimmune disease. Future studies in different patient populations are needed to define the role of TNFR2-expressing B cells in onset and progression of immune-mediated diseases and infection.

## Ethics Statement

Peripheral blood mononuclear cells (PBMC) were isolated from buffy coats from healthy donors obtained from German Red Cross South institute for transfusion medicine and immune hematology (Frankfurt am Main, Germany). The use was approved by the ethics committee of the Medical faculty of the University of Frankfurt (Approval #154/15).

## Author Contributions

Conceived and designed the experiments: OT and IB-D. Performed the experiments: OT and LM. Analyzed the data: OT, IB-D, and LM. Contributed reagents/materials: IB-D and HW. Wrote the paper: IB-D, OT, and HW.

## Conflict of Interest Statement

The authors declare that the research was conducted in the absence of any commercial or financial relationships that could be construed as a potential conflict of interest.
